# Molecular analyses identifies new domains and structural differences among *Streptococcus pneumoniae* immune evasion proteins PspC and Hic

**DOI:** 10.1038/s41598-020-79362-3

**Published:** 2021-01-18

**Authors:** Shanshan Du, Cláudia Vilhena, Samantha King, Alfredo Sahagún-Ruiz, Sven Hammerschmidt, Christine Skerka, Peter F. Zipfel

**Affiliations:** 1 Department of Infection Biology, Leibniz Institute for Natural Product Research and Infection Biology, Jena, Germany; 2 Center for Microbial Pathogenesis, Abigail Wexner Research Institute at Nationwide Children’s Hospital, Columbus, OH USA; 3 Department of Pediatrics, The Ohio State University, Columbus, OH USA; 4 Molecular Immunology Laboratory, Department of Microbiology and Immunology, Faculty of Veterinary Medicine and Animal Husbandry, National Autonomous University of Mexico, Mexico City, Mexico; 5 Department of Molecular Genetics and Infection Biology, Interfaculty Institute for Genetics and Functional Genomics, Center for Functional Genomics of Microbes, University of Greifswald, Greifswald, Germany; 6 Institute of Microbiology, Friedrich-Schiller-University, Jena, Germany

**Keywords:** Microbiology, Bacteriology, Immunology, Complement cascade, Immune evasion

## Abstract

The PspC and Hic proteins of *Streptococcus* *pneumoniae* are some of the most variable microbial immune evasion proteins identified to date. Due to structural similarities and conserved binding profiles, it was assumed for a long time that these pneumococcal surface proteins represent a protein family comprised of eleven subgroups. Recently, however, the evaluation of more proteins revealed a greater diversity of individual proteins. In contrast to previous assumptions a pattern evaluation of six PspC and five Hic variants, each representing one of the previously defined subgroups, revealed distinct structural and likely functionally regions of the proteins, and identified nine new domains and new domain alternates. Several domains are unique to PspC and Hic variants, while other domains are also present in other virulence factors encoded by pneumococci and other bacterial pathogens. This knowledge improved pattern evaluation at the level of full-length proteins, allowed a sequence comparison at the domain level and identified domains with a modular composition. This novel strategy increased understanding of individual proteins variability and modular domain composition, enabled a structural and functional characterization at the domain level and furthermore revealed substantial structural differences between PspC and Hic proteins. Given the exceptional genomic diversity of the multifunctional PspC and Hic proteins a detailed structural and functional evaluation need to be performed at the strain level. Such knowledge will also be useful for molecular strain typing and characterizing PspC and Hic proteins from new clinical *S. pneumoniae* strains.

## Introduction

### The pathobiont *Streptococcus pneumonia*

*S. pneumoniae* (the pneumococcus) is the leading cause of community-acquired pneumonia. In addition, this Gram-positive pathogen can cause otitis media and may also cause acute life-threatening invasive infections such as sepsis and meningitis^1–4^. Malnutrition and *S. pneumoniae* infections are the major cause of childhood mortality worldwide. Pneumonia accounts for approximately 16 percent of the 5.6 millions of deaths among children under five years of age, killing around 808,000 children in 2016 according to the United Nations Children’s Fund (UNICEF) and the World Health Organization (WHO)^5–7^. At any point in time pneumococci can reside asymptomatically in the upper respiratory tract of about 50% of children, from where they can spread to other sites and cause disease or be transmitted to other individuals^8^. Based on the differences in the polysaccharide capsule 100 *S. pneumoniae* serotypes have been identified so far^9,10^.

Pneumococcal diseases are widespread and antibiotic resistant strains are constantly emerging resulting in a need for new therapeutics. In addition, currently available vaccines are based on the capsular polysaccharide and only provide protection against the limited number of serotypes included. Vaccines protecting against a higher number of serotypes or a serotype-independent vaccine is needed to combat the pathogen efficiently. These limitations make it important to identify new virulence determinants that may serve as novel vaccine or therapeutic targets, to understand the diversity of these determinants and also to define the immune escape strategies of this pathogenic bacterium^1,11,12^.

Immune and in particular complement evasion is critical for all pathogenic microbes, including *S. pneumoniae*. Common mechanisms of complement evasion are emerging as a large list of pathogenic microbes bind and exploit the same human complement regulators^13–17^. Thus, it is important to understand the exact role of individual pneumococcal virulence determinants in complement and immune evasion. Furthermore, it is important to establish whether the virulence determinants are localized to the surface and, if so, the specific regions of the protein exposed^18–21^.

### PspC and Hic proteins as central pneumococcal immune evasion proteins

The PspC and Hic proteins are important pneumococcal immune evasion proteins and adhesins that represent promising vaccine candidates^22^. The majority of virulent *S. pneumoniae* strains express at least one PspC or Hic variant, and strains that have the *pspC/hic* genes deleted show significant amelioration of lung infection, nasopharyngeal colonization, and bacteremia in mice^23^.

Based on overall sequence similarities PspC and Hic variants are considered to represent one group of pneumococcal immune evasion proteins. Initial analyses by Brooks Walter in 1999 and Iannelli et al. in 2001 revealed both sequence similarity and diversity among PspC and Hic proteins^24,25^. Ianelli et al. identified several domains within the 43 PspC and Hic proteins evaluated including, the leader peptide, α-helical regions with a seven-amino acid periodicity, repeat domains and a proline-rich stretch followed by either a choline-binding or sortase-dependent anchor^26^. At that time, the cell wall anchors were used as the criterion to differentiate between PspC and Hic family members and based on sequence differences six PspC-type and five Hic-type clusters were defined. However, today there are still no precise criteria regarding cluster specific domain composition or domain characteristics. Because the patterns of domains are not exactly known and the borders of individual domains are not well-defined, a straightforward system of variant designation is at present difficult to achieve. This makes assignment of existing and newly identified *pspC* *and* *hic* genes, including those from novel clinical pneumococcal isolates, difficult or even impossible^27^.

Initially, PspC was identified as an adhesin, which targets the secretory component of secretory Immunoglobulin A (sIgA) and the polymeric IgA receptor (pIgR)^28^. Because *pspC* and *hic* genes were identified independently by multiple groups, different names were given, including CbpA (choline-binding protein A), SpsA (secretory IgA binding protein), PbcA (C3-binding protein A), or Hic (Factor H binding inhibitor of complement) (Table 1)^29–39^. Over time *pspC* and *hic* have become the favored nomenclature.

**Table 1:** Host regulators binding to *S. pneumoniae* PspC and Hic proteins.

Host regulators	Function	Binding site
Factor H	Complement regulation	HVD
slgA/plgR	Adhesion	Repeat domains
C3	C3 inactivation	Not mapped
C4BP	CP inhibition	Not mapped
Plasminogen	Proenzyme; plasmin cleaves inactivates C3, C3b and fibrin	Not mapped
Thrombospondin-1	Adhesive glycoprotein, cell–cell and cell–matrix interaction	Not mapped
Vitronectin	Complement control and adhesion	Not mapped
Lactoferrin	Fe metabolism	Proposed by homology
IgA	IgA inactivation?	Proposed by homology

The domains are listed in order of their location starting from the N-terminus. Known domains and new domains are included. The table includes domains which are found in both PspC and Hic variants, domains which are specific for either PspC or Hic, and those which are found in other bacterial proteins.

*SP* signal peptide, *HVD* hypervariable Domain, *RD* Repeat Domain, *RCD* random coil domain, *S*_*n*_*D/GS*_*2*_ Serine Rich segment, *RCE* random coil extension, *R-type* repeat related Domain; *EPRD* extracellular proline rich domain, *VS* variant specific, *IgA* IgA binding domain, *PRD* proline-rich domain, *CBP* choline-binding domain.

PspC and Hic proteins are are attached to the bacterial cell wall. PspC proteins attach non-covalently to the phosphorylcholine (PCho) moiety of teichoic acids (TAs) via their C-terminal choline binding domains and Hic proteins, are covalently linked to the peptidoglycan via an LPsTG motif. The fact that both proteins are anchored via their C-terminal regions suggests that the N-terminal region of the protein spans the capsular polysaccharides and extends beyond the capsule into the external environment. However, the different mechanisms of localization suggest that there might be differences between PspC and Hic in the strengths of interaction with the bacterial surface. Furthermore, attachment of Hic to the peptidoglycan will result in the protein being attached closer to the cell membrane.

PspC and Hic proteins bind several human plasma proteins including Factor H, C3, C4BP, Plasminogen, thrombospondin-1, and vitronectin^26,28–41^. These multifunctional proteins represent one of the most diverse group of immune evasion proteins^26,41^. PspC and Hic proteins have a mosaic structure, comprised of distinct regions that consist of multiple domains. Furthermore, a substantial overlap of domains exists between PspC and Hic variants. Standard domain or sequence-based comparisons between members of this protein family are complex due to structural differences and variable domain composition. Currently, the protein NCBI databank lists 54,852 entries for PspC or Hic and 12,193 entries for CbpA, including both full-length proteins and partial protein sequences (October 13, 2020; NCBI www.ncbi.nlm.nih.gov/protein). The individual entries show homology, but also exhibit considerable variation in structure and sequence. Examination of several PspC and Hic proteins revealed proteins composed with variable domain patterns, different combinations of domains, and novel domains.

### Mosaic-structured PspC and Hic proteins

Our understanding of these important pneumococcal immune evasion proteins is currently incomplete. Thus, our ability to understand the function of single domains, know the binding sites for host ligands, determine how the proteins of different strains vary in structure, and correlate these properties with disease states is limited. To achieve these goals it is essential to define the exact domain composition of individual PspC and Hic variants.

### Aim of the study

Thus far, the domain organization of individual PspC and Hic variants, whether each domain is likely within or extending beyond the cell wall and precise borders of the domains is unclear. Furthermore we do not know exactly which domain(s) are integrated into the bacterial cell wall, which domain(s) span the capsule and which domains are externally positioned. Given these limitations, and the heterogeneity among these important immune evasion proteins, we aimed to evaluate the structure and domain composition of six PspC and five Hic variants, each representing one of the clusters defined by Ianelli et al.^40^. We further aimed to define domain composition and position. Our studies illustrate structural and compositional differences between the full-length PspC and Hic proteins, within the PspC or Hic group and between the N and C-terminal regions. Furthermore, this comparison also identified nine new domains and several subvariants.

## Results

### Global similarity of PspC and Hic variant proteins

#### Selection of PspC and Hic variants

**Figure 1:**
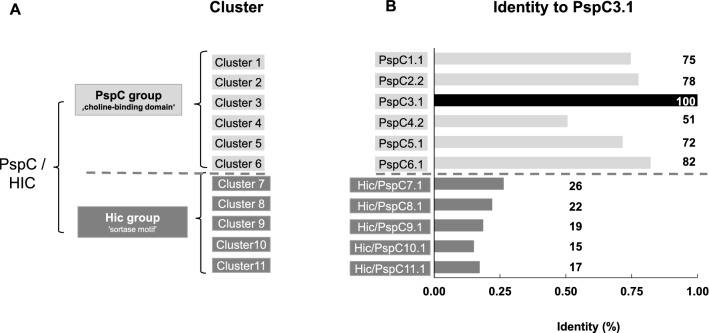
Diversity of PspC and Hic cluster variants. PspC and Hic proteins were initially considered to represent one protein class that, based on the different surface anchors, can be divided into two major groups. (**A**) PspC variants with choline-binding domains representing the PspC group, and Hic variants with sortase dependent LPsTG motifs for cell wall anchoring representing the Hic group. For each group additional clusters were identified. For the analysis one variant from each cluster was selected, i.e. for the PspC group: PspC1.1, PspC2.2, PspC3.1, PspC4.2, PspC5.1, PspC6.1; and for the Hic group: Hic/PspC7.1, Hic/PspC8.1, Hic/PspC9.1, Hic/PspC10.1, Hic/PspC11.1. (**B**) Amino acid identity of the full-length selected cluster variants with PspC3.1. The variation identified for the six PspC and the five Hic variants selected is indicative of compositional variation among the two major protein groups.

One protein from each variant cluster as defined by Ianelli et al. was selected^40^. These are the six PspC variant clusters, i.e. PspC1.1, PspC2.2, PspC3.1, PspC4.2, PspC5.1, PspC6.1, and the five Hic variant clusters, Hic/PspC7.1, Hic/PspC8.1, Hic/PspC9.1, Hic/PspC10.1, Hic/PspC11.1. At the date of the cluster designation Ianelli et al*.* considered the PspC and Hic variants as one protein family and used a PspC nomenclature for both protein groups^37^. To preserve the differentiation between Hic and PspC families and at the same time follow the nomenclature suggested by Ianelli et al*.* we combined the Hic and PspC designations (Fig. 1A). The selected proteins vary in size and mass, with PspC1.1 being the largest protein with a length of 929 aa and a molecular mass of 110 kDa, while Hic/PspC8.1 is the smallest protein with a length of 503 aa and a mass of 65 kDa (Supplementary Table I). When compared to the well-characterized PspC3.1 protein (strain D39), the overall amino acid identity of the six PspC proteins ranged from 51 to 82%. In contrast, the five Hic variants were less conserved, with aa sequence identity ranging from 15 to 26%. These high levels of sequence diversity also suggest functional differences between the PspC and Hic variants (Fig. 1B).

#### PspC3.1 as a prototype PspC

PspC3.1 was selected as a prototype and used for analyzing structure and domain composition. PspC3.1 has a signal peptide that directs the protein to export. The N-terminal region of the protein extends beyond the cell surface, while the C-terminal region interacts with the teichoic acids of the bacterial cell wall via the C-terminal Choline-Binding Domain. Because some regions of these proteins are within the cell wall while others extend beyond, we hypothesized, that hydrophilic and hydrophobic surroundings, could influence protein structure and composition.

#### Structure and residue composition of PspC3.1

**Figure 2:**
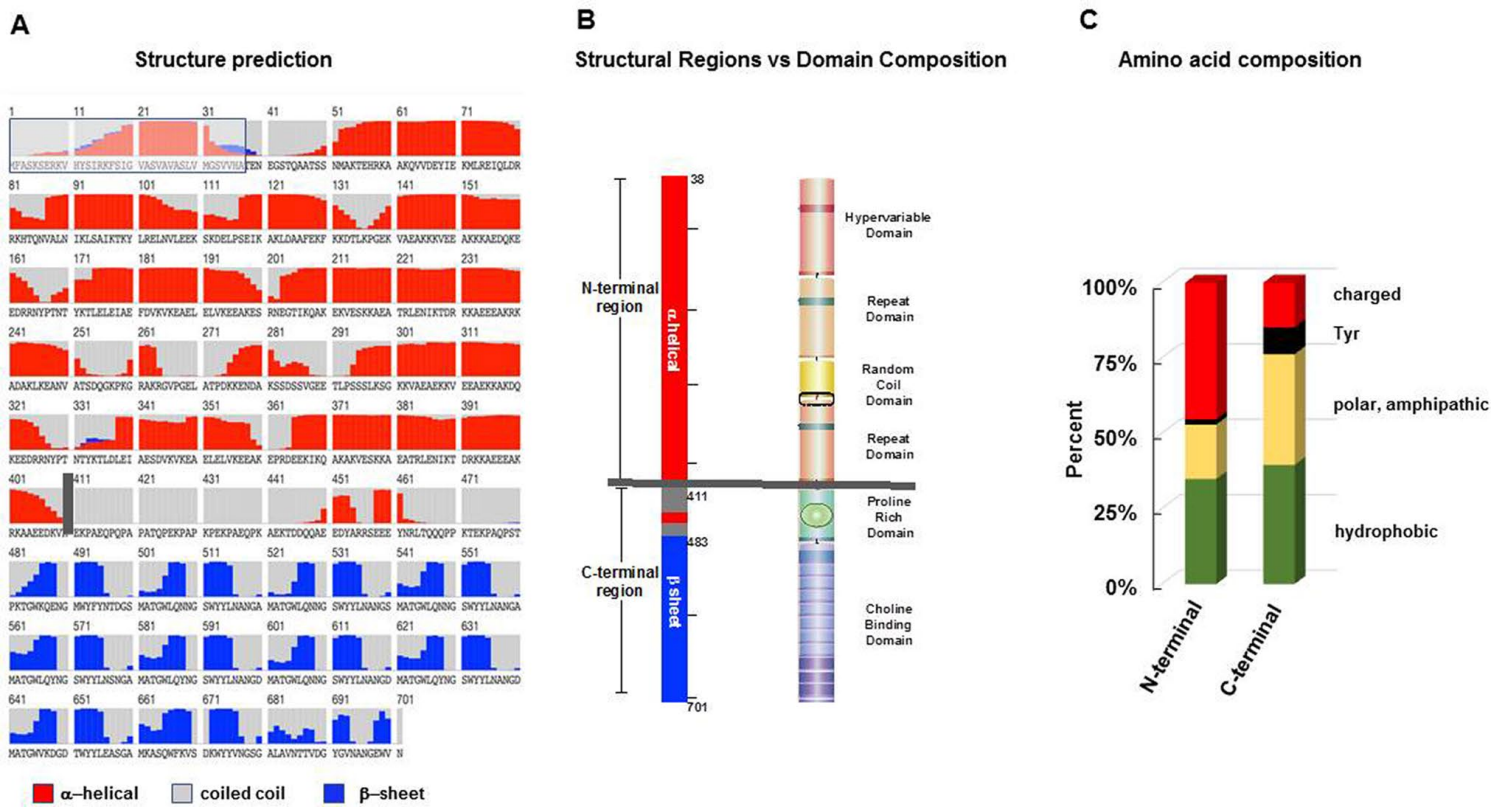
Structural regions and domain position of PspC3.1. *Dissection of PspC3.1 into distinct structural regions using *in silico analyses. (**A**) Secondary structure of the well-characterized PspC3.1 variant (strain D39). The N-terminal part of the molecule shows a long stretch composed mainly of α-helices (red bars) (aa 1–410) followed by a 72 aa coiled-coil segment (grey area) and a 219 aa region consisting mainly of β-sheet folds (blue bars). The numbers represent the amino acid position. The signal peptide (positions 1–37) which is cleaved upon processing is shown by the box with grey background and blue lines. The vertical grey bar separating the N-terminal α-helical from the coiled-coil region may represent the boundary to the bacterial cell wall. (**B**) Structural regions and domain composition of PspC3.1. The mainly α-helical region (positions 38 to 410) is termed the N-terminal region. The remainder of the protein includes the 72 aa coiled-coil and the 219 aa mainly β-sheet segments is termed the C-terminal region (left panel). To correlate structural regions with the domain composition, the known domains of PspC3.1 were included (right panel). The Hypervariable Domain, Repeat Domain I, Random Coil Domain and Repeat Domain II aligned with the N-terminal, mainly α-helical region. In the C-terminal region of the protein the coiled-coil segment consisted of the Proline-Rich Domain and the β-sheet segment with the Choline-Binding Domain. The grey horizontal line separates the N and C-terminal regions and likely marks the border of the cell wall and capsule facing the outside environment. (**C**) Amino acid composition of N and C-terminal regions. The amino acid composition was evaluated separately for each region. The N-terminal region is rich in charged residues (48%), has a low number of both polar and amphipathic residues (24%), and Tyr residues (left panel). In contrast, the C-terminal region contained a lower percentage of charged residues (22%), had more polar and amphipathic amino acids (38%) and more Tyr residues (8%).

In silico analysis of PspC3.1 revealed three different structural regions. The N-terminal 410 residues form mostly α-helices, this region is followed by a 70 aa predominately coiled-coil region and a 221 aa region composed mainly of β-sheets (Fig. 2A). Given these structural differences the 410 aa mainly α-helical region was designated as the N-terminal region and the remainder of the protein containing the coiled-coil and β-sheet segments was designated the C-terminal region.

For the purpose of this study, the terminology region is used to describe longer protein elements which have related structural or compositional features. Domains are considered to represent separate, individual folding units which display specific functions. Single domains can be further subelements, including modular elements or repeat units which are assembled in repetitive manner and which can vary in sequence and in aa length.

When the structural regions were aligned with the previously identified domains of PspC3.1, the N-terminal α-helical region included the signal peptide, the Hypervariable Domain, the two Repeat Domains, and the Random Coil Domain. The Hypervariable Domain includes the binding sites for human Factor H and each Repeat Domain includes a binding site for sIgA/polymeric Ig receptor, which is in agreement with the concept that these domains extend beyond the cell wall. In contrast the C-terminal region consist of domains expected to be within the cell wall and in the membrane. The mostly coiled-coil region represented the Proline-Rich Domain (aa 411–482), which is considered a cell wall-spanning and flexible domain and the β-sheet region represented the Choline-Binding Domain (aa 483–701) which mediates attachment to the cell wall (Fig. 2B)^41,42,55,56^.

#### Amino acid composition

Next we evaluated if the proposed cell wall integration and external environments influence the protein make up. Of the aa residues within the N-terminal region of PspC3.1 45.3% are charged, 18.0% are polar and amphipathic residues and a low proportion are Tyr (1.7**%**). In contrast, the C-terminal region contains a lower percentage of charged residues (15.0%), an increased percentage of polar and amphipathic amino acids (9.5%) and a high level of Tyr residues (8.9%) (Fig. 2C)**.** Thus, the N-terminal and C-terminal regions of PspC3.1 differ in domain structure, and amino acid composition.

#### The differences between the N and C-terminal regions are conserved in the other PspC and Hic variants

Next we evaluated if the structural composition, as outlined for PspC3.1, is conserved in the other PspC and Hic variants. The N-terminal region of all analyzed PspC and Hic variants consists mainly of α-helices, and the C-terminal Proline-Rich Domains are predominantly coiled-coil structures. The Choline-Binding Domains within the C-terminal PspC variants consist mainly of β-sheets, while the Hic specific LPsTG anchors consist of a coiled-coil stretch followed by an α-helical segment (Supplementary Figs. 1 and 2).

**Figure 3:**
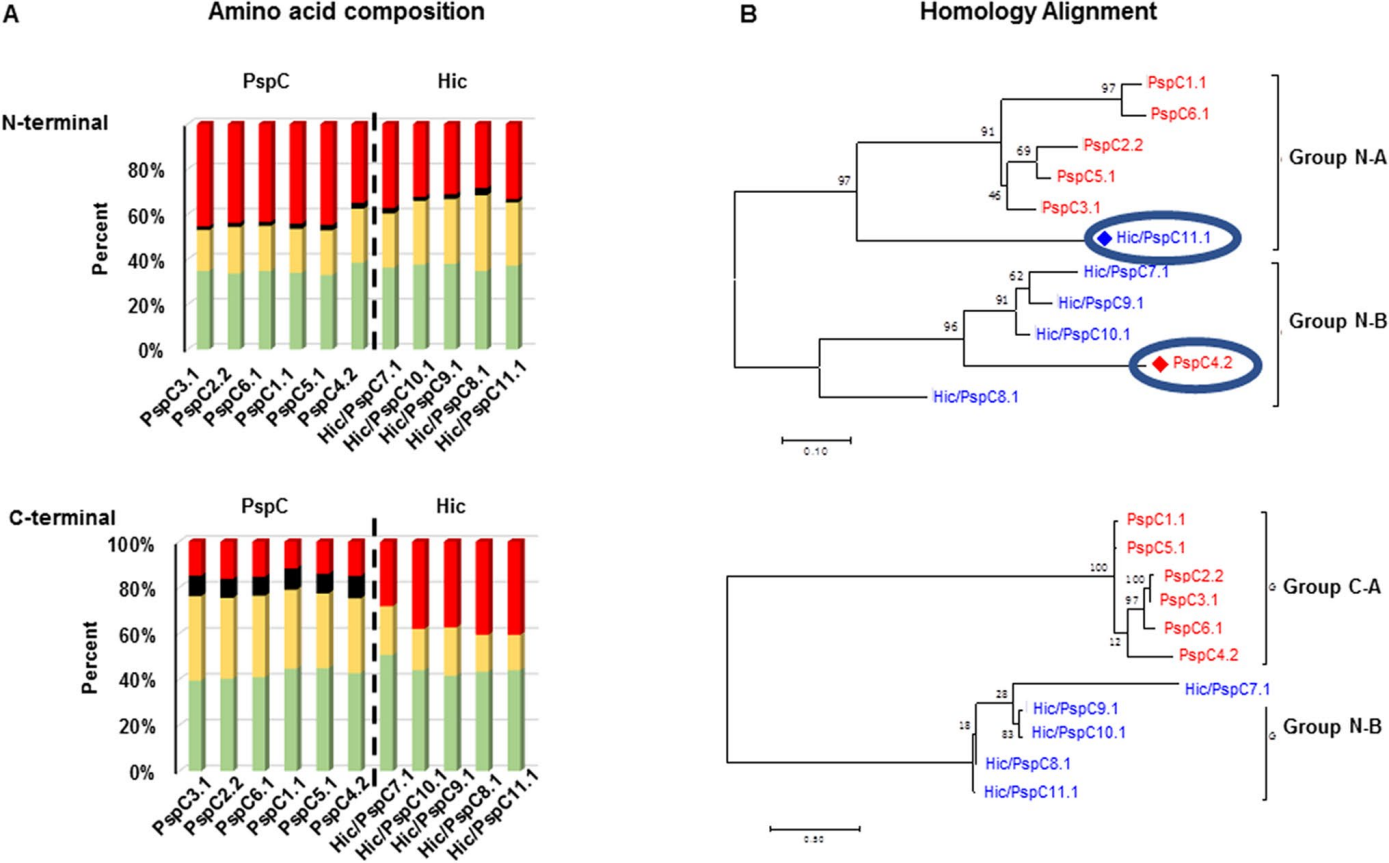
Differences in the N and C-terminal regions of the PspC and Hic variants. (**A**) The N and C-terminal regions of PspC and Hic type proteins differ in amino acid composition. The amino acid composition of the N and C-terminal regions was evaluated for each selected variant. The N-terminal regions of the six PspC and the five Hic variants are rich in charged residues (35–45%) and have a low number of both polar and amphipathic, and Tyr residues. The PspC variants had also a high proportion of charged residues (28–42%) (upper panel). The C-terminal regions of the PspC variants had a lower percentage of charged residues (16% or less) and more polar and hydrophilic (32–36%) and Tyr residues (8.3–9.1%). The composition of the C-terminal region of Hic variants differed from that of PspC variants. The C-terminal regions of Hic variants contained more charged residues, lower levels of Tyr and polar and amphipathic residues (lower panel). (**B**) Phylogenetic trees of the N and C-terminal regions of PspC and Hic type proteins. The homology alignment of the N and C-terminal regions identifies two groups. For the N-terminal regions group A is dominated by PspC type proteins, but also includes Hic/PspC11.1. Group B is dominated by Hic type proteins, but also includes the PspC4.2 variant. The C-terminal regions show a clear separation between the PspC and Hic variants.

In addition, the amino acid composition was determined. Thirty-five to forty-five percent of the aa residues in the N-terminal regions of the six PspC variants are charged. In contrast only 16% of residues in their C-terminal regions were charged. The C-terminal regions of the PspC variants also contained more polar and amphipathic amino acids (32–36%), and were rich in Tyr residues (8.3–9.8%) (Fig. 3A). Charged residues were common in both the N-terminal (28–37%) and C-terminal (28–41%) regions of the Hic variants. Furthermore, the C-terminal region of Hic variants contained less polar and amphipathic residues (15–21%) than the PspC variants (Fig. 3A). Thus, the N and C-terminal regions of the proteins differ in structure and amino acid composition, and the C-terminal regions of the PspC and Hic proteins show differences in amino acid composition.

The N-terminal regions of the different variants ranged in length from 146 (Hic/PspC8.1) to 633 (PspC5.1) residues. A homology alignment of the N-terminal regions showed two distinct clusters. One N-terminal cluster included five PspC variants (PspC1.1, PspC6.1, PspC2.2, PspC5.1, PspC3.1) and the Hic/PspC11.1 variant, while the second N-terminal panel included PspC4.2 and four Hic variants (Hic/PspC7.1, HicPspC9.1, Hic/PspC10.1, Hic/PspC8.1) (Fig. 3B, upper panel). The C-terminal regions were more conserved in length, ranging from 236 (PspC5.1) to 348 aa (Hic/PspC8.1) and by sequences clearly separated into distinct PspC and Hic groups. The level of diversity between the C-terminal regions of variants within each group was low indicating that these domains are more highly conserved (Fig. 3B, lower panel).

#### Domain analyses of PspC and Hic variants

**Figure 4:**
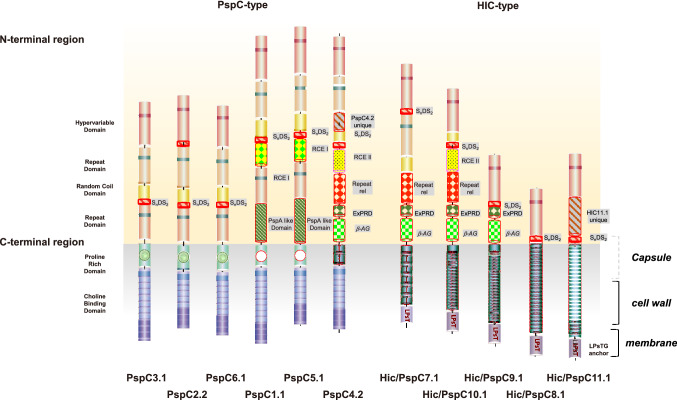
Domain structure of the six PspC and five Hic variants. The domain architecture of PspC3.1 is shown on the left-hand side. The PspC and Hic variants differ in length and domain number. The proteins are arranged based on their overall homology. To reflect the different lengths of the regions proposed to be within cell wall and the external environment the proteins are centered along the axis which separates the N-terminal, α-helical region from the C-terminal region. The N-terminal and C-terminal regions are shown on yellow and grey backgrounds, respectively. Proteins are drawn to scale. The signal peptides and for the Hic-cluster the C-terminal region which is cleaved upon anchoring are not included. Domains previously identified within PspC3.1 are shown in solid colors. New domains are patterned, and their names are given alongside the domain on a grey background. The predicted binding sites for the human plasma protein Factor H within the Hypervariable Domain are shown by the purple bar and those of sIgA/pIgR within the Repeat Domains by green bars. The PspA like domain and the b-AG binding domains were identified by homology with the binding domains within *S. pneumoniae* protein PspA and the IBC protein from *S. agalactiae*.

Using PspC3.1 with its five known domains as a blueprint, a sequence based comparison was followed to determine the presence and organization of domains within the other ten cluster variants was evaluated. This approach identified three domains of PspC3.1, the signal peptide, the N-terminal Hypervariable Domain and the C-terminal Proline-Rich Domains, present in all PspC and Hic variants. All PspC variants use a Choline-Binding Domain, while Hic/PspC proteins have an LPsTG anchor (Figs. 1 and 4). The Repeat Domains and the Random Coil Domain are found mainly in PspC proteins, but not in all variants. Additional sequences were identified in some variants that did not match known domains of PspC3.1. These domains were evaluated to determine whether they are present in other PspC and Hic variants or whether homologs exist in the protein data bank. This approach identified nine new domains, including one new domain in PspC3.1, and three new sub-variants of the Proline Rich Domain. Including these new domains in an examination of the PspC and Hic variants revealed that the individual proteins harbour between four (Hic/PspC8.1) and ten different domains (PspC4.2) (Fig. 4).

#### Known domains of the N-terminal region

The known domains identified in the N-terminal region include:

#### Signal peptide

A highly-conserved 37 aa N-terminal signal sequence which directs the proteins for export and is cleaved upon processing, is present in all PspC and Hic/PspC variants (Supplementary Fig. 3A).

#### Hypervariable domains

**Figure 5:**
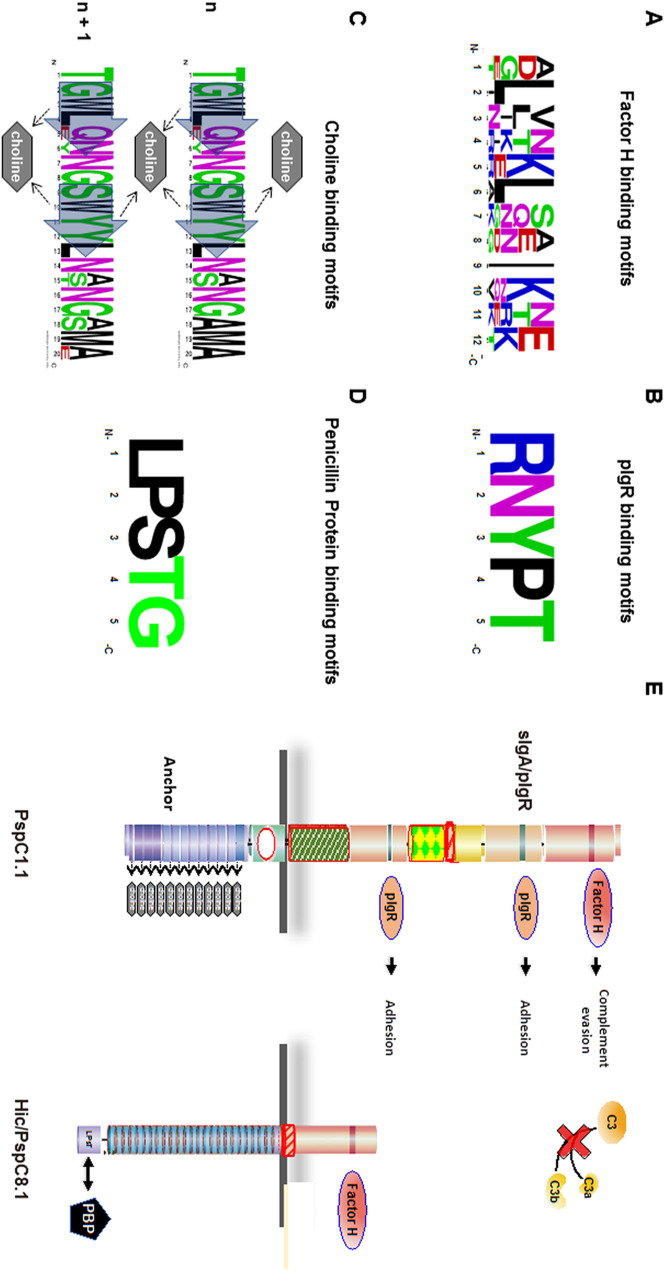
Sequence Variation and Conservation of Binding Domains and Surface Orientation of PspC1.1 and Hic/PspC8.1. (**A**) Sequence variation of the Factor H binding motif within the Hypervariable Domains of the six PspC and five Hic variants. WebLogo was used to evaluate amino acid variation. (**B**) Sequence conservation of the binding sites for human sIgA/pIgR in Repeat Domains I and II. (**C**) WebLogo was used to evaluate sequence variations I the second and third choline-binding modules of the PspC variants. Sequence variation among the Choline-Binding Modules 2 and 3 of the PspC variants. Residues relevant for the interaction with choline are indicated by the box arrows and include Trp at positon#3, i.e.W_3_ and W_10_ of module n, as well as Y_11_ of module n + 1. (**D**) Sequence conservation of the sortase recognition motifs LPsTG in the C-termini of Hic-type proteins. (**E**) Structure of and proposed orientation of the phosphorlylcholine (PCho) associated PspC1.1, and sortase A dependent covalently linked Hic/PspC8.1 variant. The arrangement is based on the concept that PspC1.1 is non-covalently associated to the teichoic acids via its interaction with PCho. In contrast the Hic/PspC8.1 variant is covalently linked via the sortase anchor to peptidoglycan Penicillin binding protein (PBP). This attachment and orientation suggests that the Proline-Rich Domains may represent a flexible cell wall and capsule spanning segment. The grey line represents the bacterial membrane and cell wall, and the capsule is indicated by the shaded grey region. The domains proposed to extend beyond the cell wall and capsule exdomains are shown in yellow or red. The binding domains for human plasma regulator Factor H within the Hypervariable Domains (PspC1.1 and Hic/PspC81) and the sIgA or cell surface receptor pIgR binding domains within the Repeat Domains I and II (PspC1.1) are indicated by purple and green bars, respectively. Attached Factor H mediates complement evasion and blocks complement mediated opsonophagocytosis and release of the anaphylatoxins C3a and C5a. SIgA or pIgR bind to two sites in PspC1.1 and block opsonization by sIgA or mediate adhesion to human epithelial cells. The binding sites of vitronectin and other human plasma proteins remain to be mapped. The C-terminal regions, with a proposed location within the cell wall or capsule are shown in green, blue or purple and include the Proline Rich Domains followed by Choline-Binding Domains(PspC1.1) or LPsTG mediated anchor (Hic/PspC8.1).

At the N-terminus of the mature Hic and PspC proteins are the Factor H binding Hypervariable Domains^26, 28^.These domains are rich in charged residues and vary in length from 91 (PspC4.2) to 113 aa (PspC2.2). As their name suggests, they were highly variable in sequence with each PspC and Hic variant examined encoding a distinct variant (Supplementary Fig. 3B). Only five residues, **T**_11_,**S**_12_,**I**_**59**_,**Y**_**63,**_**K**_**96**_ (numbering based on PspC3.1) present in all variants; although, additional residues are conserved in several variants. Factor H binding by PspC3.1 is mediated by a 12 amino acid region^28^, we identified diversity in this region of different variants and whether these domains all bind Factor H remains to be determined (Fig. 5A, Supplementary Fig. 3C).

Relationship analysis using a dendrogram identified three subtypes of the hypervariable domains. Subtype A (HVD-A**)** is present in PspC3.1, PspC5.1, and Hic/PspC11.1, HVD-B is present in PspC2.2, PspC1.1, and PspC4.2, and HVD-C is present in PspC6.1, Hic/PspC7.1, Hic/PspC10.1, Hic/PspC9.1, and Hic/PspC8.1 (Supplementary Fig. 3C).

##### Repeat domains

All PspC-type proteins and Hic/PspC7.1 possess a repeat domain of approximately 110 aas (Repeat Domain). Five PspC variants (i.e. PspC3.1, PspC2.2, PspC6.1, PspC1.1, PspC5.1) contain a second Repeat Domain. These Repeat Domains are rich in charged residues, and include conserved RNYPT motifs, which are binding sites for sIgA/pIgR (Fig. 5B, Supplementary Fig. 4). Related repeat domains identified in pneumococcal PspK (H2BJK8) share 55% aa identity with Repeat Domain I and 71.6% identity with Repeat Domain II. The solution structure of the Repeat Domain of PspC3.4 from strain TIGR4 has been solved^42^. This domain folds into three antiparallel α-helices and the YPT residues, representing the core sIgA/pIgR binding motif, are positioned in a coiled-coil loop, which separates the first and second helices. This experimentally determined structure confirms and validates our in vitro structure prediction (Fig. 2A).

##### Random coil domain

The Random Coil Domains are typically positioned downstream of the first Repeat Domain. They are approximately 30 aas in length, have a coiled-coil structure and are relatively conserved in sequence. No homologous sequences were identified in the sequence database (Supplementary Fig. 5).

### New domains of the N-terminal region

Sequences in the PspC and Hic variants that did not match known domains of PspC3.1 were also identified. A data base search for counterparts identified nine new domains, including one new domain in PspC3.1 and also three new variants of the Proline Rich Domain.

#### Serine-rich elements

Serine-Rich Elements with the overall motif S_n_D/GS_2_ were detected in all PspC and Hic variants with the exception of PspC4.2. Nine variants harbored one serine-rich element, whereas PspC2.2 contained two. These Serine-rich elements share a coiled-coil structure; but differ in their sequence and position within the protein. Serine-rich elements following the Hypervariable Domain (PspC2.2, Hic/PspC7.1, Hic/PspC9.1, Hic/PspC8.1) or the unique Hic/PspC11.1 domain have the consensus S_n_D/GS_2_ and are up to 24 aa in length. The serine-rich elements following the Random Coil Domain (PspC3.1, PspC2.2, PspC6.1, PspC1.1, PspC5.1, Hic/PspC10.1) are comprised of S_2_DS_2_, units and can be up to 18 aa long. The domain of Hic/PspC10.1 shows a variation to these common features (Supplementary Fig. 5A). The biological role(s) of these elements are as yet unknown. However, in engineered proteins, related poly-serine-rich elements are integrated as flexible linkers that separate functional, individually folding domains^43^. Interestingly the TKPET motif at the end of S_2_DS_2_ domains following the Hypervariable domains are related to the first seven residue long units found in Proline Rich Domains III and IV (see below).

### Random coil extension domains

Two new domains were identified downstream of the Random Coil Domain-S_2_DS_2_ combination of domains.

#### Random coil extension domain 1

Two proteins, PspC1.1 and PspC5.1, contain an almost identical new 83 aa domain. This domain includes several charged residues, and shares homology with RICH type domains in other proteins, including PspC Q9KK19, SpsA O33742 and IgA Fc receptor binding protein P27951 from *Streptococcus agalactiae*. These domains are predicted to be involved in bacterial adherence or cell wall binding^44^**.**

#### Random coil extension domain 2

PspC4.2 and Hic/PspC10.1 have 114 and 126 aa domains that follow the Random Coil Domain and which share moderate sequence identity. The N-terminal domain of Hic/PspC10.1 has a 37 aa extension, with the remainder of the domain being sequence similarity with the PspC4.2 domain. The biological role of this unique segment is unclear. In PspC4.2 this domain includes a long α-helical stretch that is followed by a 30 aa coiled-coil region.

#### PspA-like domain

PspC1.1 and PspC5.1 have related, new domains following Repeat Domain II. These 130 or 131 aa domains are rich in charged residues, and share 84.5% sequence identity with the A*/B element of PspA from pneumococcal strain DBL6A. The A*/B element includes a lactoferrin-binding region^45,46^, suggesting that the newly identified domains in PspC1.1 and PspC5.1 bind lactoferrin^47,48^.

#### PspC4.2 specific element

Domain pattern analysis identified an element in PspC4.2 which is positioned between the Hypervariable Domain and the Random Coil Domain. This 33 aa α-helical structured element, lacks homology to other proteins in the databank, thus its role remains unclear.

#### Repeat type domain

PspC4.2, Hic/PspC7.1, and Hic/PspC10.1 contain related 92, 82, and 68 aa domains, respectively. These mostly α-helical domains are distantly related (41.6% aa identity) to the Repeat Domains, but lack the sIgA/pIgR binding motif (RNYPT) binding motif and seem to be specific to PspC and Hic proteins.

### A new two- segmented domain

A new two-domain segment was identified in PspC4.2 and the three Hic proteins, Hic/PspC7.1, Hic/PspC10.1, Hic/PspC9.1.

#### The upstream segment

The 24–40 aa upstream segments of this domain are rich in proline residues, have a predicted coiled-coil structure, and due to their location in the N-terminal region of PspC are termed *Extracellular Proline Rich Segments*. The high Proline content may suggest a function as linker separating domains^49^. These External Proline Rich Segments lack homology to other bacterial proteins, and thus seem unique to PspC proteins.

#### The downstream elements share sequence similarity with the Fc binding domain of protein C from *S. agalactiae*

The 78 or 89 aa elements are rich in charged residues, lack proline residues, and have an α-helical structure. A blast search revealed 51.1% aa identity with an IgA binding domain within the trypsin sensitive beta-antigen of *Streptococcus agalactiae* (strain P27951/Uniprot). This protein binds the Fc region of human IgA, likely via two putative binding sequences^50^ which are also found in several other bacterial immune evasion proteins including SpsA from *S. pneumoniae*. Based on the many charged residues this IgA binding domain (pfam05062) is also named RICH (Rich In Charged residues) the proposed function of which is bacterial adherence or cell wall binding.

#### Hic/PspC11 specific element

Following the Hypervariable Domain, Hic/PspC11.1 contains a unique 102 aa α-helical domain. Related domains were identified in most Hic/PspC11 variants, but not in other bacterial proteins Thus far, the function of this domain is unknown.

### Domain composition of the C-terminal region

The C-terminal region of each variant contains a modular Proline-Rich Domain with a Choline-Binding Domain for PspC variants or an LPsTG anchor for His variants^46–49^. The C-terminal regions of the PspC and Hic proteins analyzed are relatively conserved in length (ranging from 237 aa (PspC5.1) to 348 aa (Hic/PspC8.1)). A general pattern is emerging: PspC proteins link shorter Proline-Rich Domains (57 to 77 aa) to longer Choline-Binding Domains (179 to 219 aa), while Hic proteins combine longer, Proline-Rich Domains (186 to 286 aa) with shorter LPsTG anchors (50 to 62 aa).

#### Proline-rich domains

Proline-Rich Domains have a modular structure and connect the N-terminal region to the cell wall anchor^51^. The proposed role of these domains as spanning the bacterial cell wall-spanning is consistent with the position proximal to the anchor^51,52^. Our in-silico analysis identified a modular composition and further distinct proline-rich domains, which differ in length (57 to 286 aa), modular composition, and sequence.

#### Proline-rich domain I

Five PspC variants have highly related 59 to 77 aa domains, termed Proline Rich domain I. This modular domain can consist of two (PspC1.1, PspC5.1) or three (PspC3.1, PspC6.1, PspC2.2) segments (Supplementary Fig. 7A). The N-terminal segments have Proline dominated PAPA- and PAPAP motifs and can be up to 46 aa long. The C-terminal segments include PAPAP or PAPTP motifs, are up to 19 aa long, and have a coiled-coil structure. The middle segment, present only in the domains with three segments is conserved in length (23 aa), sequence, exhibits characteristic flanking Q-residues, and is rich in charged residues. In contrast to the other two segments this segment has a predicted α-helical structure and lacks Prolines. Such Proline-Rich segments are also found in PspA^52–54^.

#### Proline-rich domain II

PspC4.2 has a unique 57 aa-long Proline-Rich Domain. This new domain includes 19 Prolines and has an internal repeated segment with the sequence TPQVPKPEAPK. To date, this new domain has been identified only in PspC proteins) (Supplementary Fig. 7B).

#### Proline-rich domain III

Hic/PspC7.1 contains a unique 186 aa-long Proline-Rich Domain which includes an N-terminal 7 aa element followed by five almost identical 31 aa repeats (KK**P**SA**P**K**P(**G/D)MQ**P**S**P**Q**P**EGKK**P**SV**P**AQ**P**GTED). Each repeat contains nine proline residues and two KKPS(A/V)P motifs. The repeats are followed by a truncated 24 aa repeat element (Supplementary Fig. 7C, D).

#### Proline-rich domain IV

 Four Hic variants harbor 247 to 286 aa, Proline-Rich Domains containing 19, 23 or 26 modules. The modules vary in type and sequence, but all include multiple 11 aa repeats, (Supplementary Fig. 8A–C). Hic/PspC10.1 and Hic/PspC9.1 contain 14 and 16 (L/P)E**K**PKPEVKP**Q**.repeats, respectively. Both Hic/PspC8.1 and Hic/PspC11.1 contain 23 copies of a (L/P)E**T**PKPEVKP**E** repeats (variant residues are displayed as white letters on a black background). In each case, these repeats are followed by one shortened repeat and a nearly identical 16 aa-long C-terminal module, which varies only at position 15 (T/P variation) (Supplementary Fig. 8D, E, F).

#### Cell wall attachment

Both PspC and Hic/PspC variants have modular domains within their C-terminal regions that we propose span the cell wall. PspC proteins bind the cell wall via modular Choline-Binding Domains in contrast, Hic proteins have shorter, 50–62 aa- anchors that include a sortase-dependent LPsTG cell wall attachment motif^55,56^.

##### PspC-type protein variants possess choline-binding anchors

PspC type variants have C-terminal Choline-Binding Domains that range in length from 178 (PspC5.1) to 248 aa (PspC1.1) and consist of modules most of which are 20 aa in length (Fig. 5C)^57^. Related Choline-Binding Domains are found in up to 15 other pneumococcal proteins, including the immune evasion protein PspA, the autolysins LytA, LytB LytC, and CbpL^57^. In the literature these modular Choline-Binding Domains are sometimes termed choline-binding modules. However, given the domain composition of full length PspC and Hic variants we prefer to term such smaller, repetitively assembled subunits as modules.

##### Hic variants have C-terminal sortase signals

The five Hic variants analyzed share C-terminal 50–62 aa anchors which contain a pentapeptide LPsTG motif. The transpeptidase, sortase A cleaves this conserved motif between the Thr and Gly residues. Subsequently the protein is covalently linked via the Thr residue to lipid II (P3 precursor) and a penicillin binding protein^58,59^ (Fig. 5D).

## Discussion

The mature PspC and the Hic/PspC proteins are heterogeneous in structural composition and in sequence. Our analysis of domains within one member of the six PspC and five Hic variants identified 13 N-terminal and three C-terminal domains, including nine new domains and three new variants of the Proline-Rich Domain. The extensive diversity is the result of different combinations of domains, several of which are present in different numbers. Domain variability is increased by distinct variants of some domains, differences in the assembly of modular elements within domains and sequence variation. This diversity results in antigenic variation, functional specialization and mechanisms of cell wall anchoring^18,20^. Three domains, the Signal peptides, the Hypervariable Domains and Proline-Rich Domains are found in all analyzed variants (Table 2). Eleven domains are found in some variants, and two domains are unique to single variants. This extensive characterization shows a different composition of the N and C-terminal regions, reveals differences between PspC and Hic variants, as well as differences in the distribution, order, number and sequence variants of domains and repeats present.

**Table 2:** Domain used by *S. pneumoniae* PspC and Hic proteins.

#	Region			Domain	Sub domains	Class	n	Module	*Structure*		Comment host ligand
1			Known	SP			11				
2	N-term		Known	HVD	HVD-A, HVD-B, HVD-C		11		α helix	PspC/Hic specific	Factor H
3			Known	RD	RD-I, RD-II, RD-III		7		α helix		sIGA/plgR
					RD-II	PspC	5		α helix		
4			Known	RCD			8		α helix		
5		1	New	S_n_D/Gs_2_	3 Positions		10		Coiled coil		
6		2	New	RCE1		PspC	2		α helix		Lactoferrin
7		3	New	RCE2			2		A helix		
8		4	New	PspA related		PspC	2		α helix	In PspA	
9		5	New	R-type			3		α helix		IgA
10		6	New	EPRD			4		α helix		
11		7	New	IgA			4		α helix	*S. agalactiae*	
12		8	New	VS4.2		PspC	1		α helix	Specific	
13		9	New	VS11.1		Hic	1		α helix	Specific	
14	C-term		Known	PRD	PRD-IA, PRD-1B	PspC	5	Modular	Coiled coil	Also in PspA	Cell wall spanning
			New		PRD-II	PspC	1	Modular	Coiled coil	?	
			New		PRD-III	Hic	1	Modular	Coiled coil	?	
			New		PRD-IV	Hic	4	Modular	Coiled coil	?	
15			Known	Anchor	CBD	PspC	6	Modular	β sheets	Several	Anchor
16			Known		LPsTG	Hic	5		Coiled coil	Many	Anchor

The binding sites for Factor H has been mapped within the Hypervariable Domain of PspC3.1 and that of sIgA and the extracellular domain of pIgR to the RNYPT motif of Repeat Domains I and II. C3, C4BP, Plasminogen, Thrombospondin 1, vitronectin have been shown to bind intact *S. pneumoniae* and full length PspC and Hic proteins, but their binding sites have not been mapped to specific domains. Binding of Lactoferrin and IgA is proposed based on the homology between PspC and Hic variants and the *S. pneumoniae* immune escape protein PspA and the sIgA binding protein of *S. agalactiae.*

### Variability among PspC and Hic/PspC-variants

PspC, and Hic-type variants have related domains in their N-terminal regions but differ more in their C-terminal regions. The proteins have different C-terminal anchors. PspC proteins with the Choline-Binding Domains contact multiple choline-moieties in a non-covalent manner. In contrast the LPsTG anchors attach the proteins covalently to the peptidoglycan^56^. The type of C-terminal anchor not only influences cell wall attachment, but the length and composition of the Proline-Rich Domains. Furthermore the cell wall anchors seems to influence selection, composition, and number of the N-terminal domains. These differences in structure likely alter the role of the proteins in immune evasion and may result in different domains extending beyond the cell wall.

### Variability of N vs C-terminal regions

Broadly speaking, each PspC and Hic protein is divided into two major parts: the N-terminal region that extends beyond the cell wall and includes immune evasion and adhesion domains, and the C-terminal anchoring region.

The N-terminal regions of the PspC and Hic proteins analyzed vary in length, and domain number, ranging from 155 aa containing two domains (Hic/PspC8.1) to 610 aa containing eight domains (PspC4.2). These regions share structural features, including long α-helical structures, and a high proportion of charged residues. The Hypervariable Regions are most likely located most distant from the cell surface and show the highest degree of sequence variation. This diversity can reflect differences in antigenic variability, which is relevant for evading immune recognition by antibodies. Six of the N-terminal domains are unique to PspC and Hic variants, others like the PspA Related Domain and the region with homology to the IgA binding β antigen are found in other bacterial immune evasion proteins (Supplementary Fig. 6C).

The C-terminal regions are more conserved in length, have more polar and amphipathic residues and in the case of PspC variants also have more Tyr residues. The Proline-Rich Domains, preceding the PspC and Hic-specific anchors, are of variable length, have a modular composition, consist mostly of coiled-coil structures. Proline-Rich Domains of PspC proteins are shorter than those of Hic proteins. Given the proposed location at the interface between cell wall and capsule, such diversity could result in different binding dynamics, strength of cell wall integration, morphological differences or capsule thickness^53–62^. Similarly, the anchor domains in the C-terminus differ in length, composition, and type of cell wall attachment.

#### Protein orientation, and cell wall integration

PspC and Hic are cell wall associated surface proteins and we are starting to understand which regions of the proteins are spanning the cell wall and capsule, and which might be extended into the environment. The N-terminal region, by extending beyond the capsule, is exposed to the external environment and can interact with human proteins. The C-terminal region includes a capsule spanning region and an internal cell wall anchor.

Cell wall attachment via the C-terminal anchor orients the N-terminus to the external environment allowing interactions with host plasma proteins and cell receptors. An illustration of the orientation, spatial organization and known binding sits for human plasma regulators of one PspC and one Hic/PspC variant is presented in Fig. 5E. PspC1.1 is an eight domain variant that binds choline and the short four domain Hic/PspC8.1 variant have different compositions both in the N- and C-terminal regions. The variable lengths of the N-terminal regions mean these domains extend with different distances into the external environment. In a linear model, for example, Factor H, when bound via the hypervariable domain inhibits C3b formation and assists in C3b inactivation remote from the bacterial surface. Similarly, the variable length of the Proline-Rich Domains and the type of cell wall anchors encoded can result in differences in the strength of interactions and different localizations within the cell wall.

#### Tactical positioning and immune evasion

The two distinct anchors have different structures. Choline-Binding Domains are composed mainly of β-sheets, whereas sortase A dependent LPSTG anchors mainly consist of coiled-coil and α-helical structures. This not only dictates whether cell wall attachment is non-covalent or. covalent, but is also indicative of a more flexible vs. fixed cell wall interaction. These distinctions in cell-wall attachment may result in a different surface distribution and likely the extent to which the protein is exposed to the external environment. Indeed, different spatial localization of the PspC and Hic/PspC variants both expressed by *S. pneumoniae* strain BNH418, was shown by super resolution microscopy^63^. The PspC-protein, with the Choline-Binding Domain localized to the division septum and Factor H, when bound to this protein, controlled C3b opsonization. In contrast, the LPsTG anchored Hic protein was localized to the bacterial poles. Such differences in surface localization could influence the site on the bacteria where complement control and adhesion to host cells occurs. Therefore, these differences in distribution can influence the biological function of these important immune evasion proteins.

When comparing prevalence and distribution of PspC and Hic variants among 349 pneumococcal isolates from adult patients with invasive pneumococcal disease, 298 isolates (85.4%) had a single *pspC*-variant, 22 isolates had a (6.3%) a *hic*-variant, 19 isolates (5.4%) had *pspC* and *hic* gene and only 10 isolates (2.9%) did not possess either gene^64^. In addition, invasive, PspC expressing strains bound more Factor H, and Factor H binding and immune control was more effective in encapsulated as compared to unencapsulated strains. Similarly, the PspC variants PspC2 and PspC6 were more efficient in Factor H binding and complement inhibition on the bacterial surface as compared to the Hic variants, Hic/Pspc9 and Hic/PspC11^65,66^.

#### Conclusions and perspectives

Evaluating the domain composition of selected PspC and Hic variants and an in-depth characterization of the domain composition advanced our understanding of the structure of these virulence determinants. Our approach identified differences between PspC and Hic proteins beyond their distinct membrane anchors. Such knowledge allows a comparison of full-length proteins based on domain patterns, numbers and can result in a better comparison of different PspC and Hic/Hic variants. Similarly, individual domains can be compared based on structure, modular composition and sequence.

Analyzing the additional > 60,000 PspC and Hic proteins deposited in the NCBI protein database or gene products from additional clinical isolates, will likely identify additional variants due to the discovery of new domains and subdomains, and novel domain combinations. Defining the diversity within these pneumococcal virulence factors will increase understanding of their role in immune evasion and provide important information for molecular strain typing and vaccine design. Finally, this may also allow a correlation between PspC or Hic type variants with invasive pneumococcal infections and with clinical outcome.

## Materials and methods

### Selection of PspC and Hic variant proteins

Each of the selected six PspC and five Hic proteins represent one of the two clusters as initially defined by Ianelli et al.^40^. The sequences were derived from the NCBI database (status: Feb 2018). The PspC/Hic designation is based on Iannelli et al.^40^. The protein names, corresponding bacterial strain, protein size, GenBank Accession number and protein ID are shown in (Supplementary Table I).

#### Secondary structure evaluation

The structure (α-helical, coiled-coil and β-sheet) of each selected PspC and Hic protein was evaluated using RaptorX (http://raptorx.uchicago.edu/http://raptorx.uchicago.edu/). PspC3.1 most predicted structural similarity 2vyuA (*p* value: 3.39e−10 and secondary structure: 42% α-helical, 43% coiled-coil and, 14% β-sheets). Analysis of the other ten PspC / Hic variants revealed a similar secondary structure (Supplementary Figs. 1–2). Each of the six PpsC variants was most similar to 2vyuA. Hic/PspC7.1, Hic/PspC8.1, Hic/PspC9.1, Hic/PspC10.1, Hic/PspC11.1) were most similar to 1w9rA, 4k12B, 2m6uA, 6iaA, 2m6uA, respectively. The secondary structure prediction are shown in the form of histograms which were constructed using ggplot2 from the R/Bioconductor.

#### Phylogenetic analysis

The PspC and Hic amino acid sequences and composition were evaluated using MEGA7 (www.megasoftware.net). There was a total of 976 positions in the final dataset^67^. The CLUSTALW program and the BLOSUM amino acid matrix were used to compare the allelic variants of PspC, following which phylograms were generated using the Neighbor-Joining method (Bootstrap value: 100). The phylogram for each domain was generated using the same method. Phylogenetic trees are modified in MEGA7.

#### Domain homology searches

BLASTp was used to identify related proteins or protein segmetns within the GenBank database available at the National Center for Biotechnology Information (http://www.ncbi.nlm.nih.gov/). Furthermore, BLAST targeting database UnipRotKB reference proteomes plus Swiss-Prot was used to find regions of local similarity between sequences (https://www.uniprot.org/blast/). All the domains in this work have been done a blast.

## Supplementary information

Supplementary Figures.

Supplementary Tables.
