# Supplementary material for: Molecular analyses identifies new domains and structural differences among Streptococcus pneumoniae immune evasion proteins PspC and Hic
Source: Sci Rep. 2021 Jan 18;11:1701. doi: 10.1038/s41598-020-79362-3 (PMC7814132; doi:10.1038/s41598-020-79362-3)
Supplement: Supplementary file 1 — Supplementary Figures. [file 41598_2020_79362_MOESM1_ESM.pptx]

## Slide 1
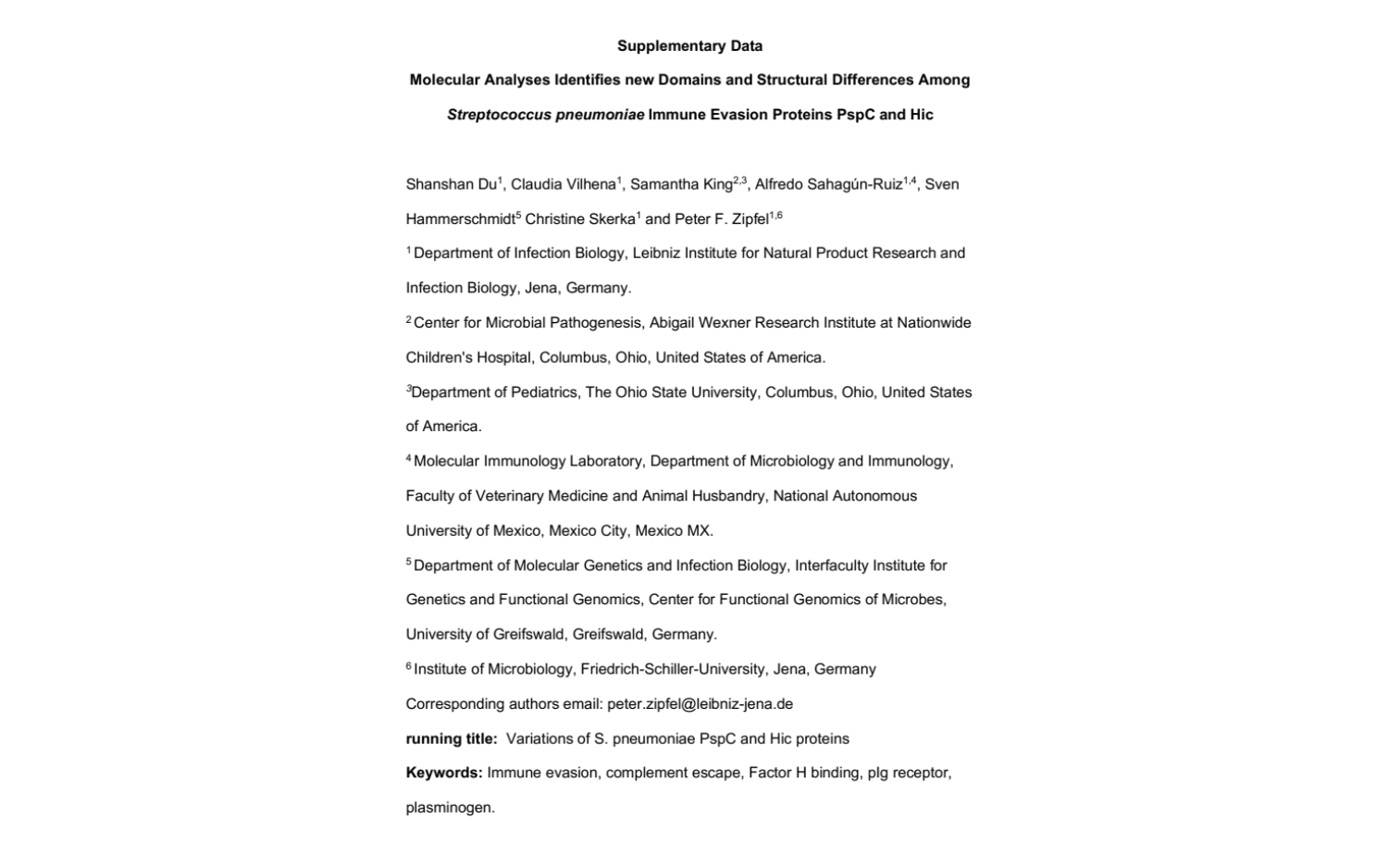

## Slide 2
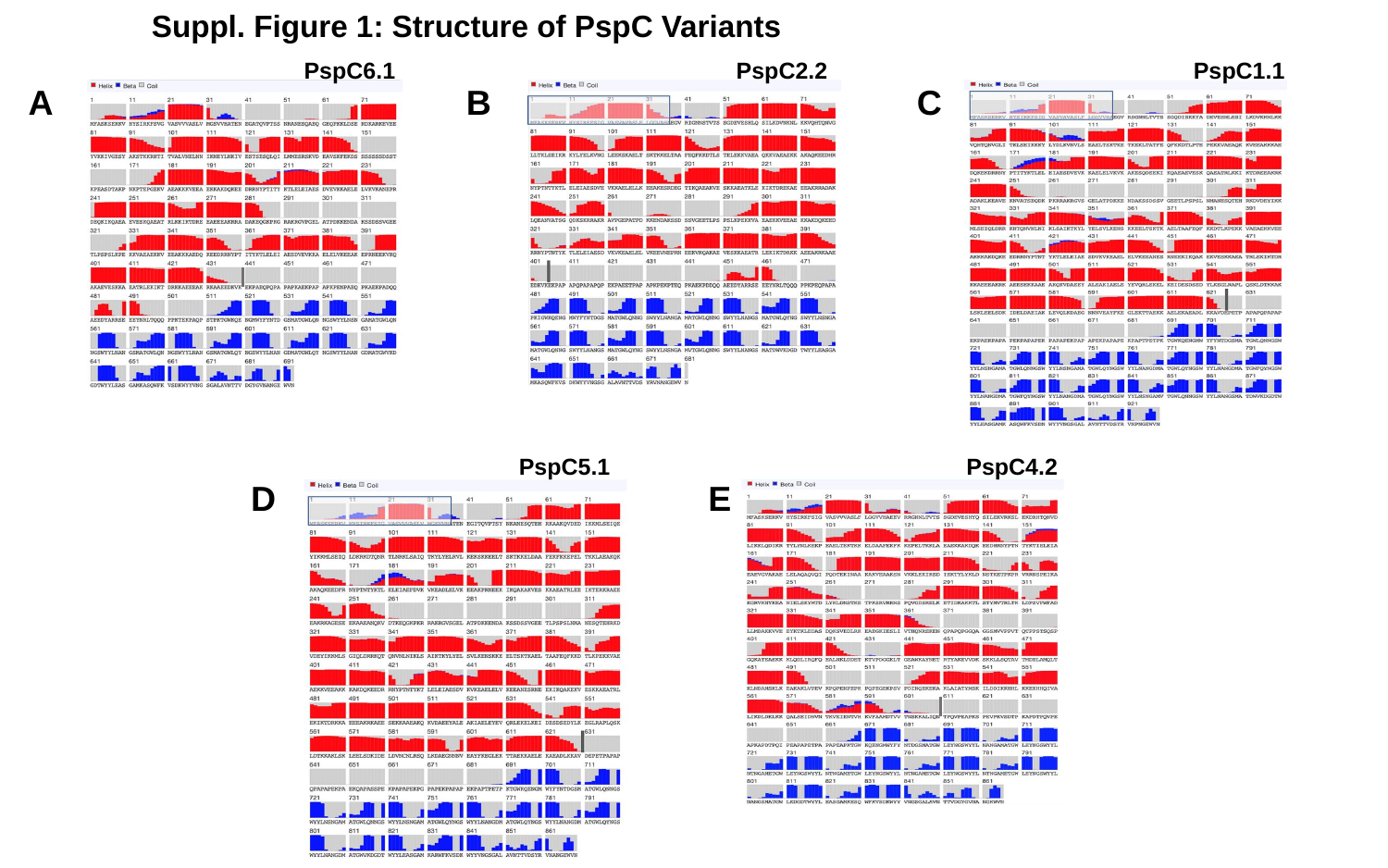

Suppl. Figure 1: Structure of PspC Variants
PspC6.1
PspC2.2
PspC1.1
A
B
C
PspC5.1
PspC4.2
D
E

## Slide 3
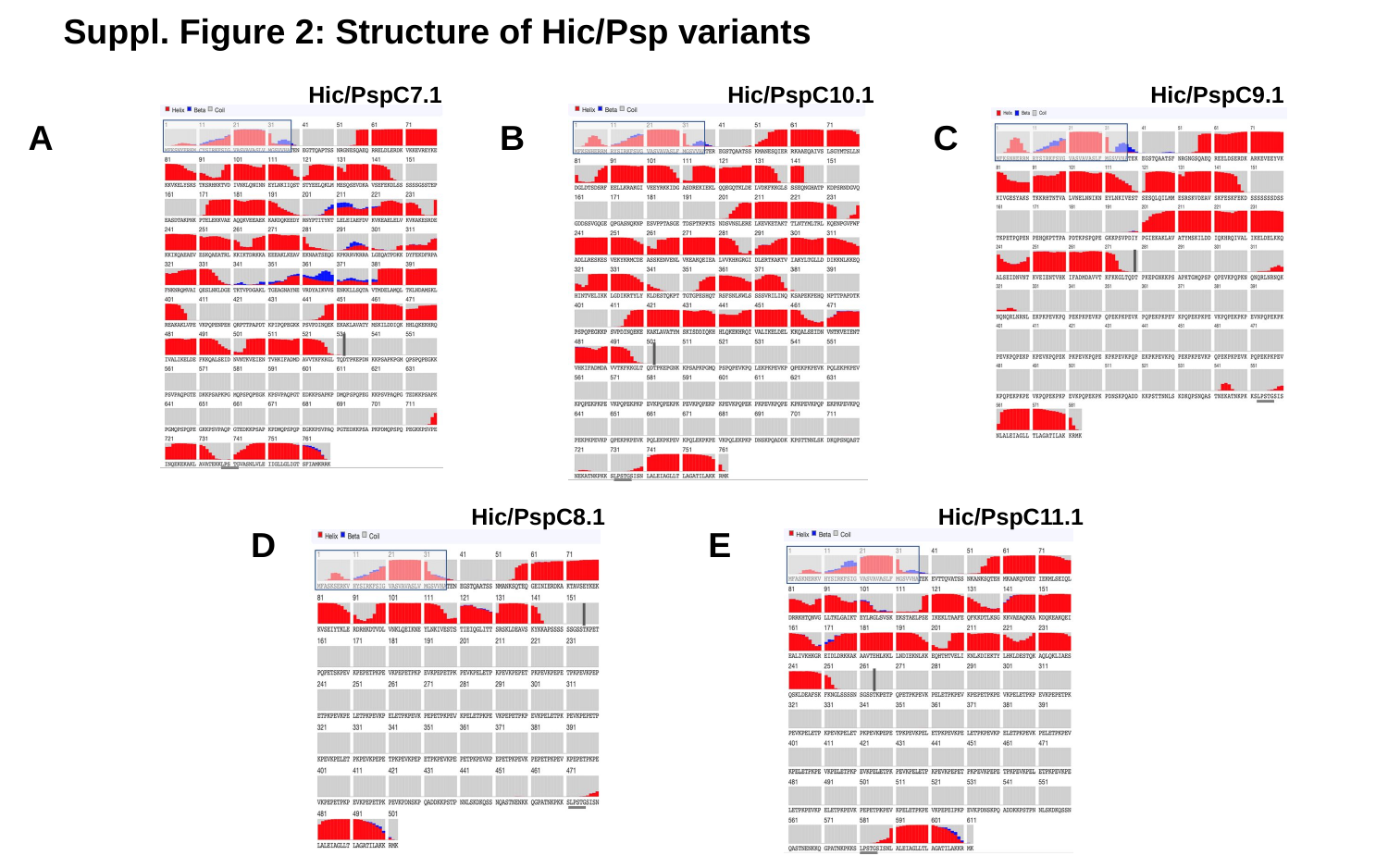

Suppl. Figure 2: Structure of Hic/Psp variants
Hic/PspC7.1
Hic/PspC10.1
Hic/PspC9.1
A
B
C
Hic/PspC8.1
Hic/PspC11.1
D
E

## Slide 4
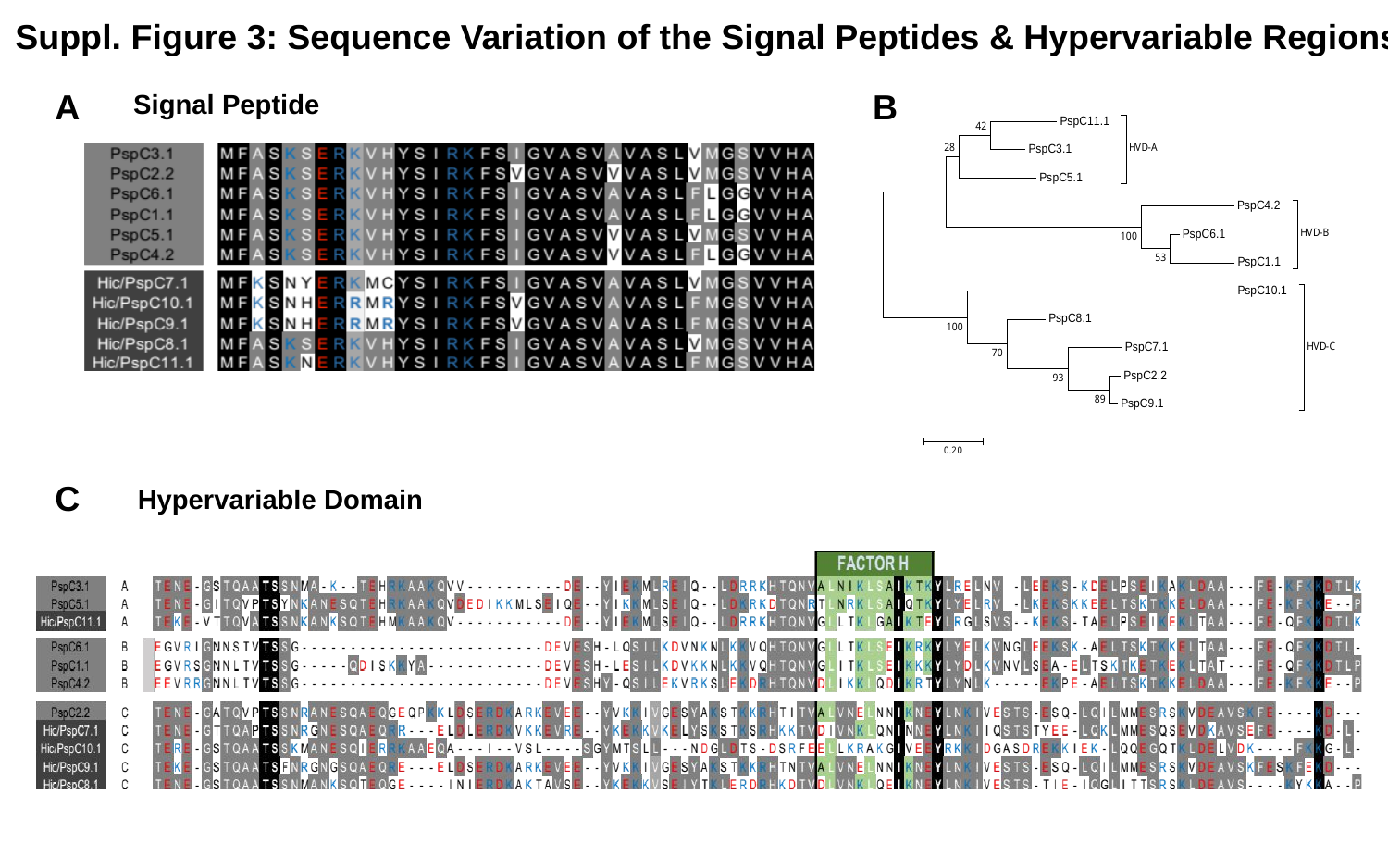

Suppl. Figure 3: Sequence Variation of the Signal Peptides & Hypervariable Regions
A
B
Signal Peptide
C
Hypervariable Domain

## Slide 5
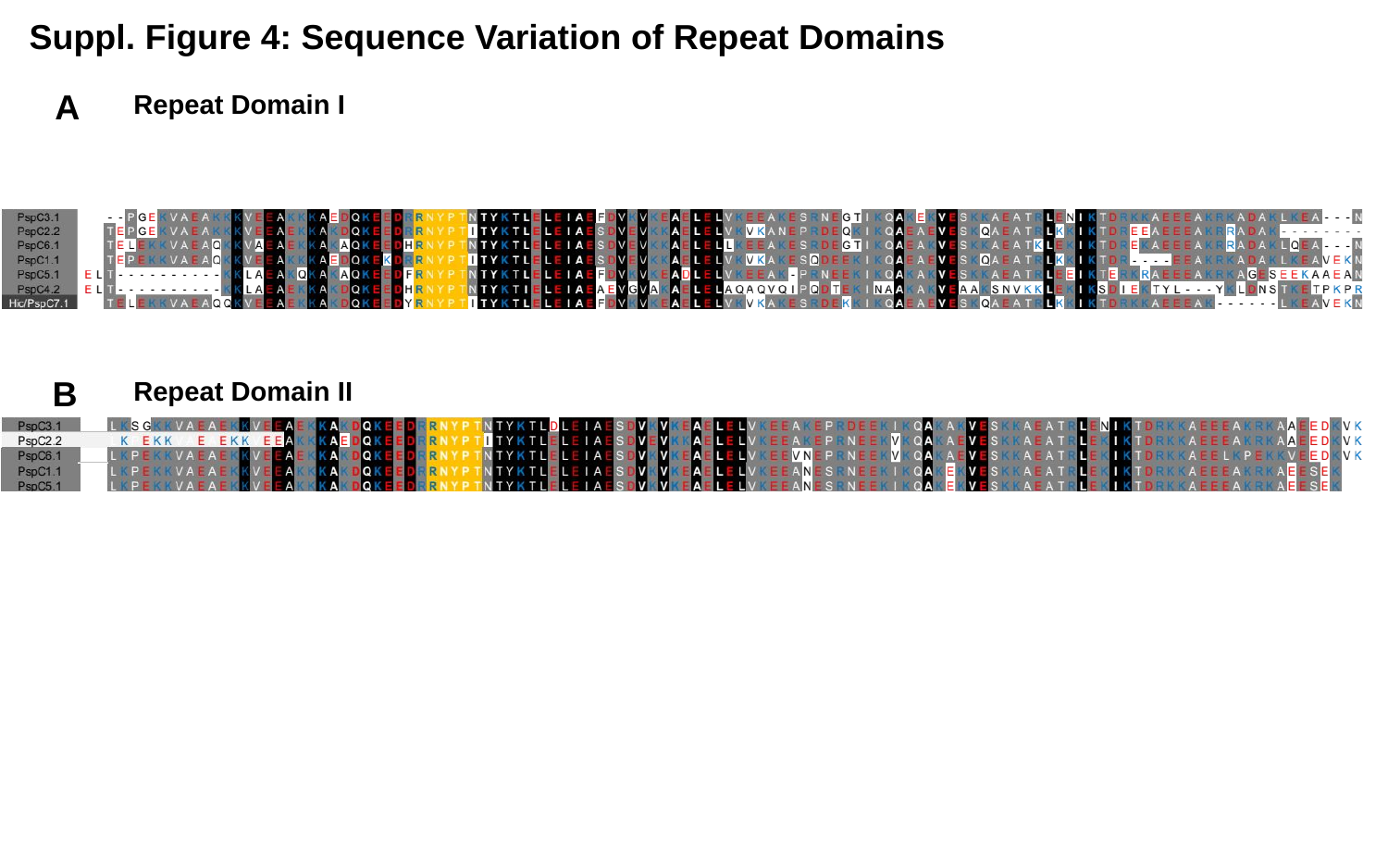

Suppl. Figure 4: Sequence Variation of Repeat Domains
A
Repeat Domain I
B
Repeat Domain II

## Slide 6
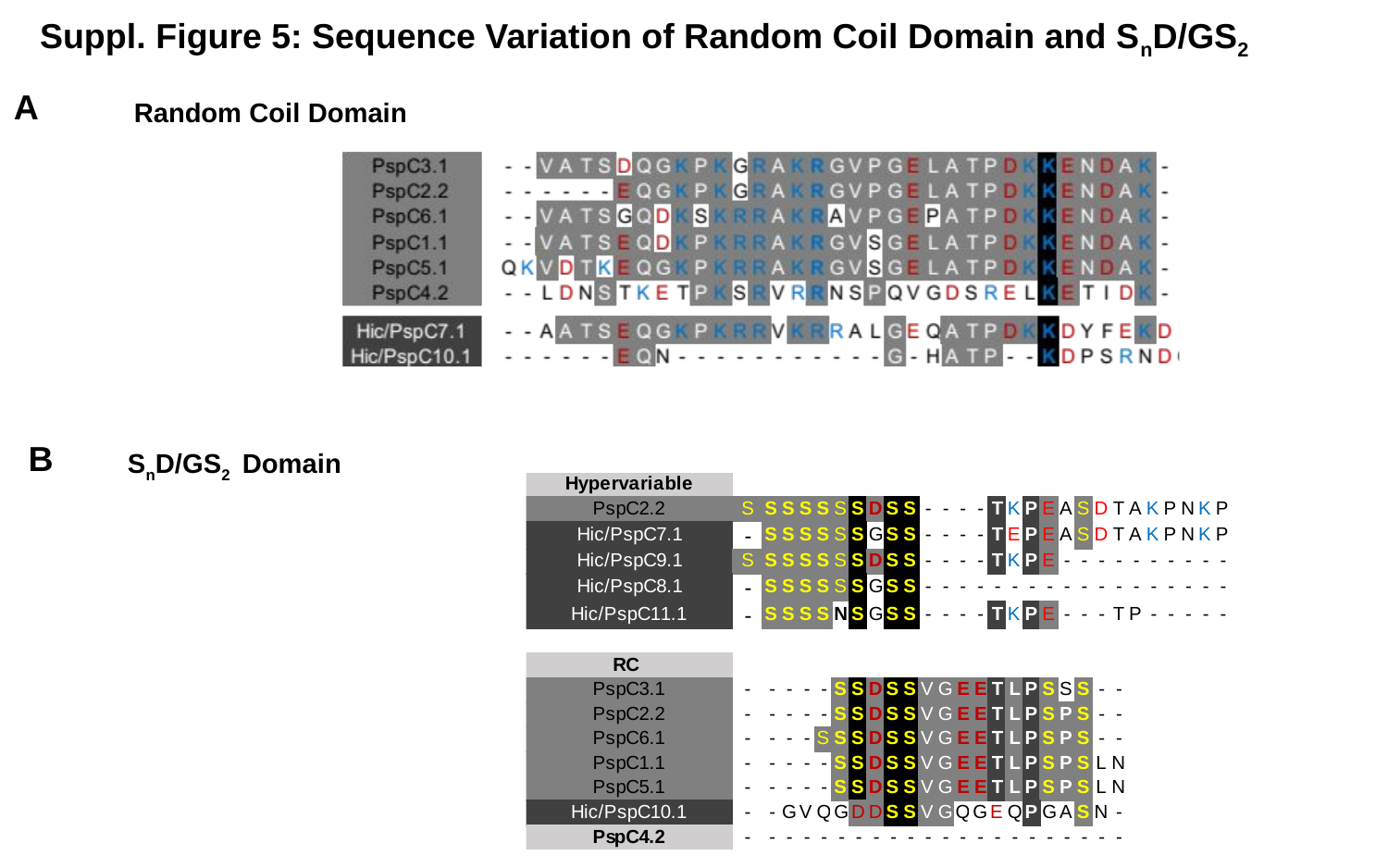

Suppl. Figure 5: Sequence Variation of Random Coil Domain and SnD/GS2
A
Random Coil Domain
B
SnD/GS2 Domain

## Slide 7
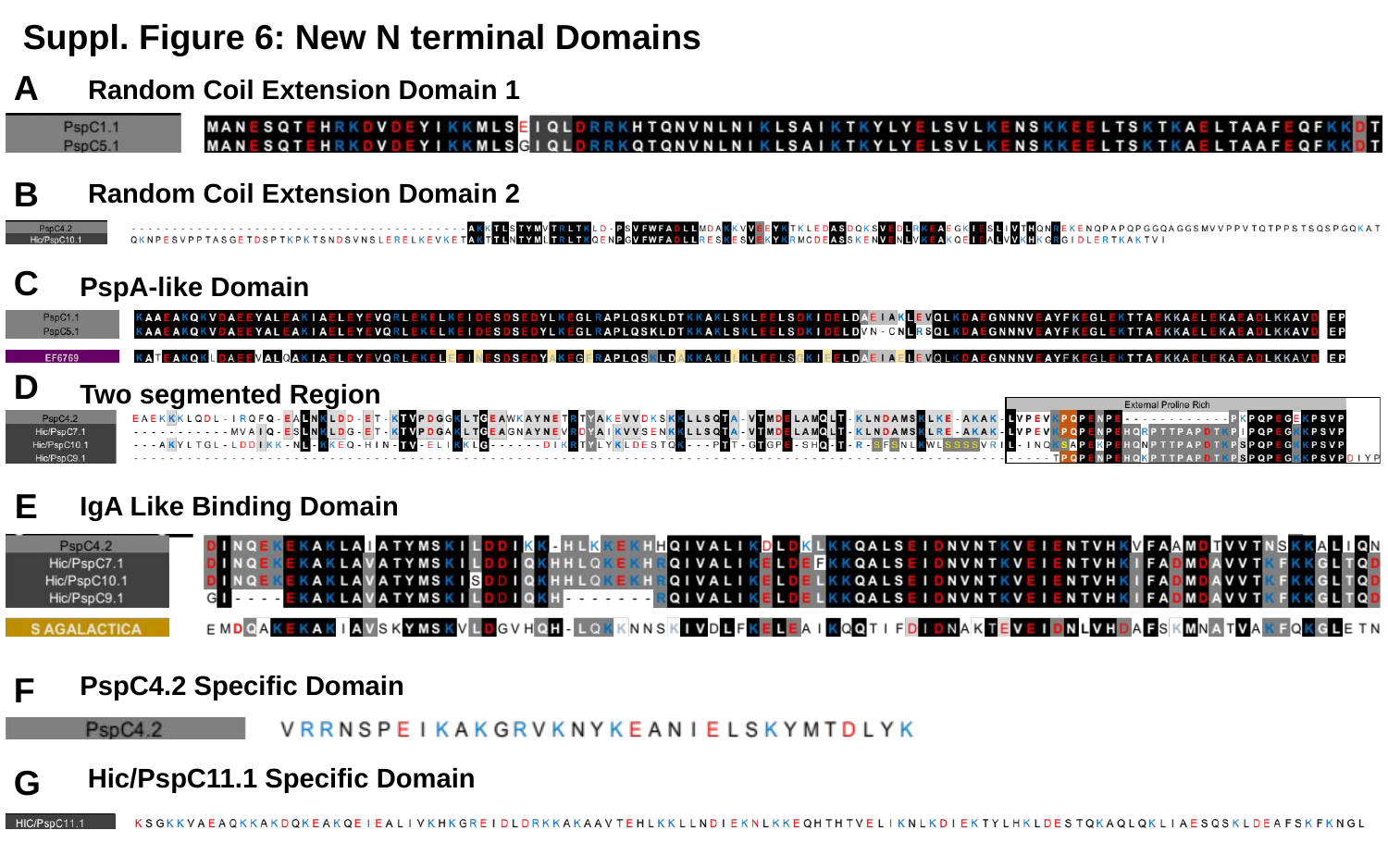

A
Repeat Domain I
Suppl. Figure 6: New N terminal Domains
A
Random Coil Extension Domain 1
B
Random Coil Extension Domain 2
C
PspA-like Domain
D
Two segmented Region
E
IgA Like Binding Domain
F
PspC4.2 Specific Domain
G
Hic/PspC11.1 Specific Domain

## Slide 8
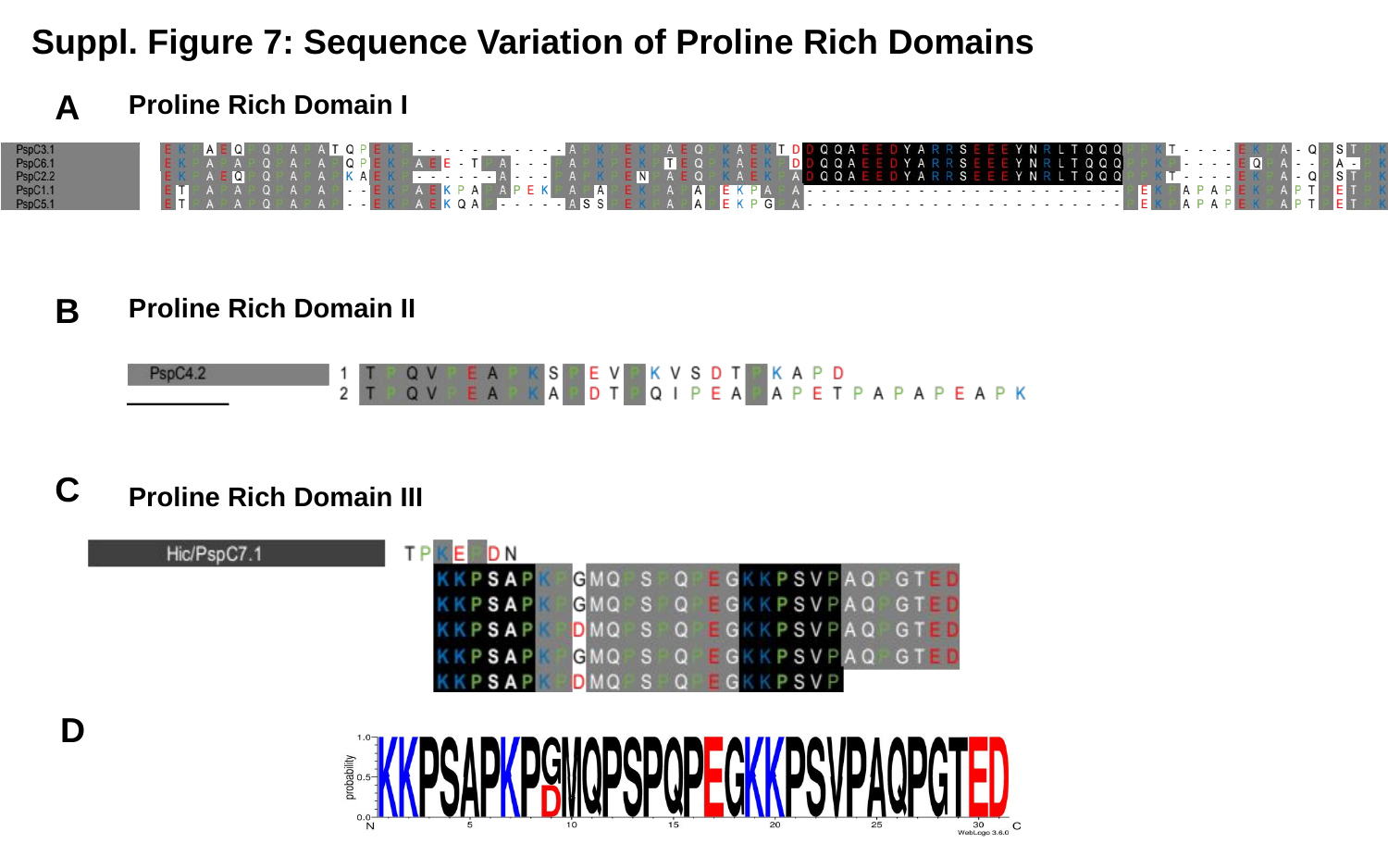

Suppl. Figure 7: Sequence Variation of Proline Rich Domains
A
Proline Rich Domain I
B
Proline Rich Domain II
C
Proline Rich Domain III
D

## Slide 9
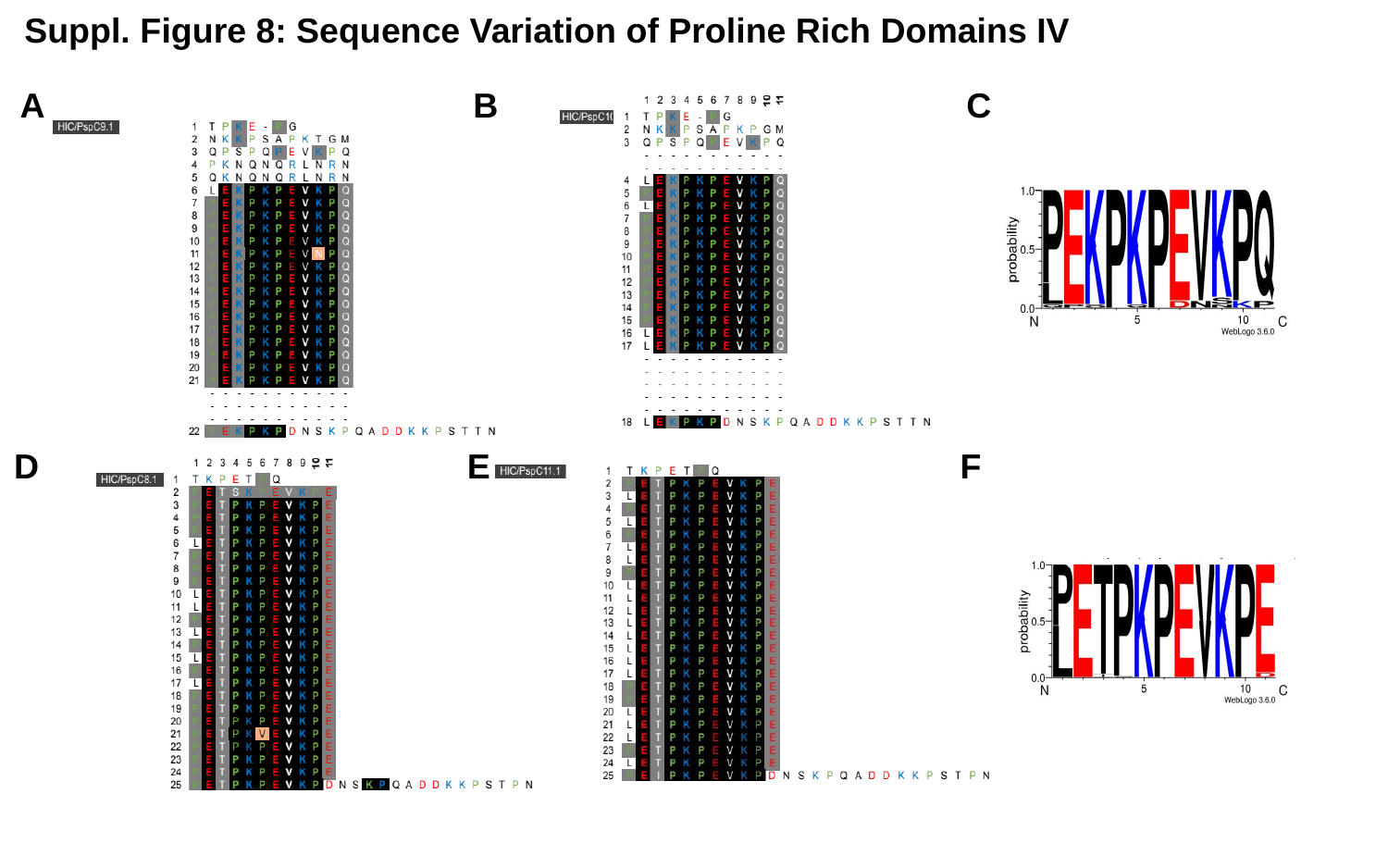

Suppl. Figure 8: Sequence Variation of Proline Rich Domains IV
A
B
C
D
E
F

## Slide 10
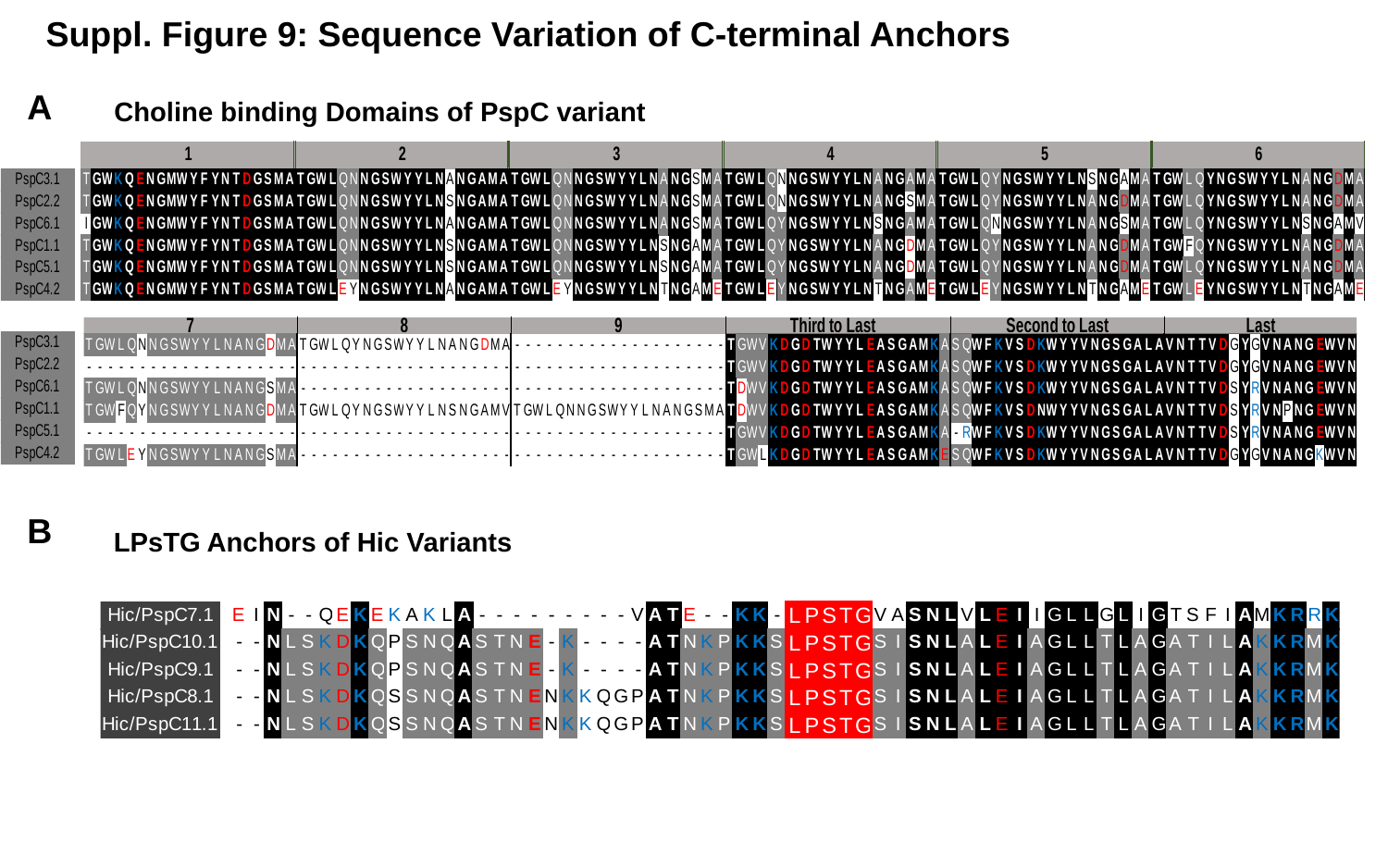

Suppl. Figure 9: Sequence Variation of C-terminal Anchors
A
Choline binding Domains of PspC variant
B
LPsTG Anchors of Hic Variants
