# Supplementary material for: Molecular analyses identifies new domains and structural differences among Streptococcus pneumoniae immune evasion proteins PspC and Hic
Source: Sci Rep. 2021 Jan 18;11:1701. doi: 10.1038/s41598-020-79362-3 (PMC7814132; doi:10.1038/s41598-020-79362-3)
Supplement: Supplementary file 2 — Supplementary Tables. [file 41598_2020_79362_MOESM2_ESM.pdf]

Supplementary Table I

Protein	Strain	Length (aa)	Genbank Accession number	Protein ID
PspC1.1	SRF10	**929**	AF154037.1	AAF73809.1
PspC2.2	G9	693	AF154035.1	AAF73807.1
PspC3.1	D39	701	AF154012.1	AAF73779.1
PspC4.2	G100	866	AF154033.1	AAF73802.1
PspC5.1	G4	869	AF154032.1	AAF73801.1
PspC6.1	G31	681	AF154044.1	AAF73817.1
Hic/PspC7.1	G54	769	AF154034.1	AAF73804.1
Hic/PspC8.1	G375	**503**	AF154015.1	AAF73783.1
Hic/PspC9.1	G31	584	AF154044.1	AAF73821.1
Hic/PspC10.1	G100	763	AF154033.1	AAF73803.1
Hic/PspC11.1	G48	612	AF276620.1	AAL90445.1
